# What the Eyes Hear: An Eye-Tracking Study on Phonological Awareness in Emirati Arabic

**DOI:** 10.3389/fpsyg.2020.00452

**Published:** 2020-03-17

**Authors:** Alexandra Marquis, Meera Al Kaabi, Tommi Leung, Fatima Boush

**Affiliations:** Department of Cognitive Sciences, United Arab Emirates University, Al Ain, United Arab Emirates

**Keywords:** neurolinguistics, Arabic language, phonological awareness, eye-tracking, word recognition, Emirati Arabic (EA), adults

## Abstract

Phonological awareness is the ability to perceive and manipulate the sounds of spoken words. It is considered a good predictor of reading and spelling abilities. In the current study, we used an eye-tracking procedure to measure fixation differences while adults completed three conditions of phonological awareness in Emirati Arabic (EA): (1) explicit instructions for onset consonant matching (OCM), (2) implicit instructions for segmentation of initial consonant (*SIC*), and (3) rhyme matching (RM). We hypothesized that fixation indices would vary according to the experimental conditions. We expected explicit instructions to facilitate task performance. Thus, eye movements should reflect more efficient fixation patterns in the explicit OCM condition in comparison to the implicit *SIC* condition. Moreover, since Arabic is consonant-based, we hypothesized that participants would perform better in the consonant conditions (i.e., OCM and *SIC*) than in the rhyme condition (i.e., RM). Finally, we expected that providing feedback during practice trials would facilitate participants’ performance overall. Response accuracy, expressed as a percentage of correct responses, was recorded alongside eye movement data. Results show that performance was significantly compromised in the RM condition, where targets received more fixations of longer average duration, and significantly longer gaze durations in comparison to the OCM and *SIC* conditions. Response accuracy was also significantly lower in the RM condition. Our results indicate that eye-tracking can be used as a tool to test phonological awareness skills and shows differences in performance between tasks containing a vowel or consonant manipulation.

## Introduction

Phonological awareness is the ability to perceive and manipulate the sounds of language (e.g., Goswami and Bryant, 1990; Holm et al., 2008). It emerges from acquired implicit phonological and lexical knowledge (Rvachew et al., 2017). In addition, it is a strong predictor of literacy (i.e., reading and spelling) skills in children (e.g., Bird et al., 1995; Schneider et al., 2000; Rvachew, 2007; Holm et al., 2008; Melby-Lervåg et al., 2012). Phonological skills in kindergartens have been found to be associated with spelling skills a year later (e.g., Speece et al., 1999). Moreover, phonological awareness deficits are characteristic of dyslexia resulting in poor reading skills (Bruck, 1992; Elbeheri and Everatt, 2007).

Rvachew et al. (2017) used a phonological awareness task where covert responses (i.e., not requiring any spoken responses or explicit articulation of speech) were analyzed so that children’s performance would be assessed independently of their speech accuracy. Children’s performance in their task ranged from random guessing to perfect performance and could be used (alone or with their other oral tasks) to predict writing difficulties (Rvachew et al., 2017). Previously, Schneider et al. (2000) found a link between phonological awareness and literacy, but their participants’ literacy better improved when phonological awareness was combined with letter-sound training. In adults, Metsala et al. (2019) found that phonological awareness skills of English-speaking adults did contribute to reading accuracy. A recent study comparing children learning to write in either English, French, German, Dutch, or Greek revealed that phonological awareness influences reading abilities differentially for the different languages studied (Landerl et al., 2019).

Phonological awareness skills and spelling abilities appear to be correlated in Arabic, as demonstrated by Taibah and Haynes (2011) and Rakhlin et al. (2019) with (Saudi) Arabic-speaking children, and Mannai and Everatt (2005) with Bahraini Arabic-speaking children. Recent results from Asadi and Abu-Rabia (2019) show that Arabic-speaking children were better at phonemes in the initial versus final position and significantly improved from the second year of kindergarten to the third, while direct comparisons between consonants and vowels/rhymes were not performed. Moreover, phonological training has been shown to improve reading skills in Arab dyslexic children (Layes et al., 2015, 2019) as well as in neurotypical Arab children (Dallasheh-Khatib et al., 2014) among other metalinguistic skills. Another study by Makhoul (2017) found that phonological awareness skills training to be implicated in improving reading development in Arab first graders and especially in at-linguistic risk groups.

The linguistic situation in the Arab world could be considered slightly complex given the diglossic nature of Arabic, which is exhibited in two distinct forms. On the one hand, there is the literary form, referred to as *Modern Standard Arabic* (MSA), also known as “fusha,” which is used by educated people in order to read and write as well as in formal communications, interviews, newspapers and other formal settings. On the other hand, there is the second form, the spoken form or dialect, which is known as “ammia” and used by speakers for daily conversations and informal settings. While MSA is more generally understood by all Arabic speakers, dialects can differ greatly from one region to another. Previous studies proposed that these two forms are couched in two cognitive systems of Arabic-speaking children and adults. For example, Saiegh-Haddad (2008) and Asaad and Eviatar (2014) conducted studies on letter learning and suggested that letters that correspond to sounds that do not occur in the Arabic dialects are more difficult to learn and to identify than the ones that do exist in the spoken Arabic variety of the speakers. Moreover, Tibi and Kirby (2018) pointed out that the diglossic situation of Arabic could pose a potential challenge in learning to use the language, since ammia, or dialectal Arabic is considered a child’s first language (Shendy, 2019) and precedes their exposure to literary MSA in most cases. Children learning to read and write for the first time in MSA may face difficulties coping with a linguistic system that is essentially foreign (Taha, 2013; Horn, 2015). One study found that reading to pre-literate children in MSA familiarizes them with the literary language (Feitelson et al., 1993). Moreover, preschoolers who were exposed to MSA were found to have better reading comprehension in subsequent years in comparison to those only exposed to the spoken dialect of Arabic (Abu-Rabia, 2000; Leikin et al., 2014). Furthermore, studies by Saiegh-Haddad (2003, 2004) found that children were better able to isolate phonemes found in their spoken dialect in comparison to those only found in literary MSA. Additionally, phonological structures found only in MSA but not in spoken Arabic dialects were less likely to be recognized (Saiegh-Haddad, 2012).

Arabic words, at least under the root-based approach, are analyzed in terms of a consonantal root, a vocalic template and other affixes (if any), all of which are considered to be independent morphological units (e.g., McCarthy, 1979, 1981; Prunet et al., 2000; Idrissi et al., 2008; among others). The consonantal roots in Arabic convey the core meaning of the word. MSA has six phonemes to represent vowels: three short vowels: /a/, /u/, and /i/ which may optionally appear in written text; and three long vowels: /a:/, /u:/, and /i:/. It is worth noting that words in MSA always begin with a consonant followed by a vowel. Alamri (2017) used eye-tracking to investigate co-activation effects of phonology, semantics, and shared roots in Arabic in the presence of competitors to the target word and concluded that roots are fundamental morphological units in the Arabic mental lexicon separate from phonology and semantics.

Arabic is described as having a consonant-based orthography (Tibi and Kirby, 2018). Arabic writing, when fully expressed (i.e., when all diacritical marks are present), may be considered to be orthographically transparent (Saiegh-Haddad and Henkin-Roitfarb, 2014), where the written form maps directly to spoken language. It is important to note that this orthographics style is not typical of texts intended for skilled adult readers who often read unvowelized Arabic text. Asaad and Eviatar (2014) proposed a further characteristic of Arabic orthography which is the presence of visual and phonological neighbors among its letters. That is, many Arabic letters share the same basic form and only differ by the number and/or the placement of dots. See examples in Table 1 below as it appears in Asaad and Eviatar (2014).

**TABLE 1:** Examples of visual and phonological neighbors in Arabic (Source: adapted from Asaad and Eviatar, 2014).

Arabic script	IPA
**Visual neighbors**	
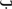	/b/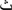	/θ/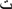	/t/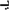	/j/

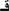	/q/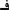	/f/

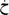	/x/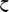	/ħ/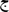	$/\hat{d}Ʒ/$

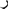	/r/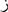	/z/

**Phonological neighbors**	

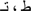	/t^ʔ^/, /t/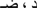	/d^ʔ^/, /d/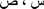	/s/, /s^ʔ^/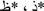	*/ð/, */ð^ʔ^/

The phonemes marked with a (*) were not in the original paper by Asaad and Eviatar (2014) as they are not present in the Palestinian Arabic dialect. They do however, exist in both MSA and Emirati Arabic.

Emirati Arabic is the dialect spoken in the United Arab Emirates. While MSA has 28 consonant phonemes (e.g., Amayreh, 2003), EA includes 30 consonant phonemes (e.g., Al Ameri, 2009). The differences between MSA and EA are that EA includes the voiced velar plosive /g/, the voiced /x/ and voiceless /ɣ/ velar fricatives as well the voiceless postalveolar affricate /tʃ/. In addition, compared to MSA, EA lacks the emphatic voiced dental plosive /d^ʕ^/, the voiced /ʁ/ and voiceless /χ/ uvular fricatives.

Measuring eye movement data has an added advantage over traditional measurements of attention such as reaction time (RT) data, as eye movements are governed by an automatic process that does not impose additional operational requirements alongside the primary task goals. In most cases, eye movements allow us to gain a “naturalistic,” yet indirect, understanding of the cognitive processes associated with task completion moment-by-moment. Therefore, we consider eye-tracking measurements as an additional index to where attention is directed moment by moment and perhaps even a superior one, as it eliminates potential confounds of recording responses mechanically.

Eye-tracking research relies heavily on what is known as the “eye-mind” hypothesis (Just and Carpenter, 1976a, b): the assumption that the direction at which the eyes are fixated at any given time is where attention, and therefore information processing, is actively taking place. Objects falling within the immediate visual field are the subject of direct attention and are therefore under active cognitive processing (e.g., Pickering et al., 2004; Conklin and Pellicer-Sánchez, 2016). Moreover, the duration of a gaze also reflects the associated processing demands on that object, such that as soon as an object is no longer fixated, it no longer requires further processing. However, it is now understood that at least for more cognitively demanding tasks, such as understanding complex sentence structures, some information processing can occur even after the text being comprehended is not being fixated foveally (Rayner et al., 2016). Therefore, it is assumed that different task demands will result in differential fixation patterns depending on the cognitive load associated with the task. In general, average fixations during free viewing of visual scenes tend to last approximately 330 milliseconds (Conklin and Pellicer-Sánchez, 2016). However, this number varies according to the task being completed. Items requiring reduced cognitive processing receive shorter fixations than those that require higher cognitive processing loads (Conklin and Pellicer-Sánchez, 2016).

Apart from reading studies (see Rayner, 2009 for a review), eye-tracking has been increasingly implicated in behavioral experiments including choice tasks (e.g., Orquin and Mueller Loose, 2013; Krajbich, 2018; for reviews), data visualization and perception of various displays (e.g., Bylinskii et al., 2015; Lahrache et al., 2018), as well as speech perception and comprehension (e.g., Tanenhaus and Trueswell, 2006). The latter area of research, and most relevant to the current study, is associated with what is known as the *Visual World Paradigm* (VWP), where “on each trial the participants hear an utterance while looking at an experimental display. Participants’ eye movements are recorded for later analyses” (Huettig et al., 2011). In such experiments, a visual display is coupled with spoken utterances. Eye movements are recorded, as they are thought to be influenced by participants’ goal for completing a particular task (e.g., Salverda et al., 2011; Pyykkönen-Klauck and Crocker, 2016; Salverda and Tanenhaus, 2017), integrating a spoken utterance with a visual display.

The current study of phonological awareness in Emirati Arabic (EA) was conducted using the VWP as the experimental method for spoken word recognition (e.g., Cooper, 1974; Tanenhaus et al., 1995; Allopenna et al., 1998; Altmann and Kamide, 1999; Richardson and Spivey, 2000; Spivey and Geng, 2001; Altmann, 2004; Knoeferle and Crocker, 2007; Ferreira et al., 2008; Richardson et al., 2009; Huettig et al., 2011). The VWP is now widely considered as a standard protocol to investigate the interplay between linguistic and visual information processing. Since the seminal work by Cooper (1974), it has been established that there exists an interactive relation between the locus of eye fixation and spoken language in the sense that the latter somehow controls the former in a time-logged fashion.

A series of work (e.g., Tanenhaus et al., 1995; Allopenna et al., 1998) further detailed a particular version of the VWP commonly used by many subsequent work. In such a version, line drawings of four objects (usually arranged on a 2 × 2 grid) are shown on a computer screen. Upon receiving audio instructions, participants have to move the displayed object by clicking on the computer mouse and dragging it to the location of a fixed geometric shape (e.g., after hearing the sentence “Pick up the beaker. Now, put it below the diamond.”). The objects displayed usually include the target (which is sometimes called the “referent”) and three distractors. Among the distractors, some are competitive in that they share some linguistic properties with the target (e.g., same onset syllable as in “candle” vs. “candy”). The primary objective of this particular configuration is not only to test how fast subjects move their eye gaze to the correct target, but also how the distractors may shed light on the language processing system vis-à-vis word recognition/identification. The distractors must therefore be carefully chosen depending on the type of language processing task targeted (e.g., phonological, semantic or pragmatic).

In an influential experiment conducted by Allopenna et al. (1998), who focused on the real time phonetic processing within the VWP given a particular target (e.g., “beaker”), the first distractor was an “onset competitor” (e.g., “beetle”), the second one was a “rhyme competitor” (e.g., “speaker”), and the third one was unrelated (e.g., “carriage”). As the spoken linguistic message started to unfold, participants demonstrated comparable fixations on the objects that shared the same onset (e.g., “beaker” and “beetle”). As more phonetic cues were presented to the participants, fixations on the onset competitor started to decline, and interestingly, fixations on the rhyme competitor (e.g., “speaker”) started to increase. This dynamic shift of fixation provides insights to the word recognition model such as the Cohort model (Marslen-Wilson, 1987), the TRACE model (McClelland and Elman, 1986), and the Shortlist model (Norris, 1994).

The purpose of many researchers when studying phonological awareness is to determine the link between phonological awareness and literacy (e.g., Holm et al., 2008; Rvachew et al., 2017; Landerl et al., 2019). The current research aims at exploring the feasibility of using eye-tracking to assess phonological awareness in EA speaking adults using a variant of the VWP in which the entire assessment tool was designed and delivered using the local EA dialect as opposed to MSA. The current phonological awareness assessment is an extension of the phonological awareness task used in the Language Acquisition Test for Arabic (LATFA, Marquis, 2016–2018); an i-Pad delivered assessments of oral literacy skills in EA children. The LATFA phonological awareness assessment comprises three phonological conditions which are expanded on below.

(1) Explicit instructions for onset consonant matching (OCM): participants are introduced to a cartoon character preceding the following example statement “This animal likes things that start with the same sound. The sound that it likes is /s/. Which of these things does it like? a. /0ʔarnab/ b. **/s**ə**kiin/** c. /ʕəriiʃ/ d. /nemer/.”
(2) Implicit instructions for segmentation of initial phoneme (*SIC*): participants are introduced to a cartoon character preceding the following example statement “This is Dana. Dana likes things that begin with the same sound as her name. Which of these things does Dana like? a. /ħaywaan/ b. /**dallah**/ c. /xað^ʕ^ ra/ d. /ʕət^ʕ^ər/.”
(3) Rhyme matching (RM): participants are introduced to a cartoon character preceding the following example statement “This is Lulu. Lulu likes things that sound like her name, which of these things is the one that Lulu likes? a. /ʔ**ubuu**/ b. /mət^ʕ^ar/ c. /dənya/ d. /mənaz/.”

The current study employs the same task conditions above, adapted for compatibility with an eye-tracker. The use of eye-tracking may allow us to gain further insight on the implicit knowledge associated with phonological awareness. Successful validation of the use of eye-movements on adults will allow us to use this task with children in order to evaluate their phonological awareness skills, which can be translated into a well-developed user-friendly mobile application used to assess phonological awareness skills. We hypothesized that fixation indices would vary across the experimental conditions relative to the easiness of each task as reflected in gaze patterns: targets in the easier tasks should receive shorter, fewer fixations and fewer visits to the interest region of the target. We expected explicit instructions to facilitate participants’ performance on the task. Thus, we predicted that eye movements would reflect more efficient fixation patterns in the OCM condition in comparison to the *SIC* condition. Moreover, since Arabic is consonant-based, we hypothesized that participants would perform better in the two consonant conditions (i.e., OCM and *SIC*) in comparison to the rhyme condition (i.e., RM). Additionally, we expected to find the same pattern of results in the accuracy data. Finally, we expected that providing feedback during practice trials would facilitate participants’ performance overall.

## Materials and Methods

### Participants

Fifty-nine female native speakers of EA took part in the experiment (Age_M_ = 20.09 *SD* = 2.12). All participants were students enrolled at United Arab Emirates University. Data from six additional participants were excluded from the analyses because they spoke a dialect of Arabic other than EA. Of the remaining participants, half received automatic, online corrective feedback during the practice trial phase (Feedback group), while the other half received no feedback (No Feedback group). All participants reported at least 18 years of permanent residence within the United Arab Emirates, and spoke the native EA dialect. None of the participants reported to have prior identified speech, hearing or visual impairments.

### Stimuli

**FIGURE 1:**
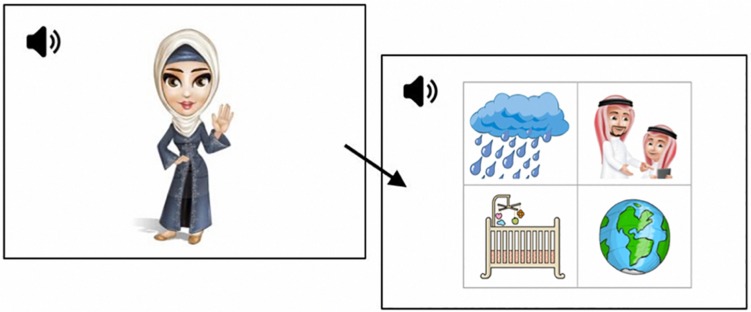
An example of a trial. **Screen 1:** “This is Lulu. Lulu likes things that sound like her name. Which of these things is the one that Lulu likes?’ **Screen 2:** /ʔubuu/ ‘father’ (target) and distractors: /met^ʕ^ar/ ‘rain’, /dənja/ ‘world’, /mənaz/ ‘cradle’.”

The task is an adaptation of the French Test de Conscience Phonologique Préscolaire (Brosseau-Lapré and Rvachew, 2008, modeled on the phonological awareness test by Bird et al., 1995), developed for EA. Images used for the current experiment were adapted from the stimuli set used for the LATFA phonological awareness assessment (Marquis, 2016–2018), with minor substitutions for items that became heavily pixelated due to re-scaling on the monitor screen used for eye-tracking. In cases where images were replaced, the new ones were either identical, or as visually similar to the originals as possible. The Emirati Arabic Language Acquisition Corpus (EMALAC, Ntelitheos and Idrissi, 2017) was used as a reference for highly familiar nouns for the construction of the images to ensure participants’ familiarity with the items and to avoid saliency effects. In total, there were 192 images arranged in combinations of four for the VWP task (see Figure 1). No image was repeated more than twice throughout the experiment, and never for the same phoneme or phonological condition. Auditory stimuli consisted of verbal task instructions in EA. All auditory stimuli were recorded by a native female EA adult. There were 4 different target phonemes for each of the three conditions, each were tested on three separate trials embedded within different targets. Each trial began with a script associated with a cartoon character whose name served as a probe for the target phoneme. There was a script for each target phoneme, where each script introduced the nature of the task. An example for each condition is given below.

(1) OCM condition: 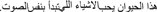 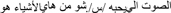 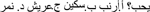  “This animal likes things that start with the same sound. The sound that it likes is /s/. Which of these things does it like? a. /ʔarnab/ ‘rabbit’ **b. /s**ə**kkiin**/ **‘knife’** c. /ʕəriiʃ/ ‘palm house’ d. /nəmər/ ‘tiger’.”
(2) *SIC* condition: 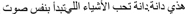 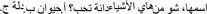 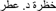  “This is Dana. Dana likes things that begin with the same sound as her name. Which of these things does Dana like? a. /ħaywaan/ ‘animal’ b. /**dallah/ ‘flask’** c. /xað^ʕ^ ra/ ‘vegetables’ d. /ʕət^ʕ^ər/ ‘perfume’.”
(3) RM condition: 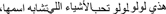 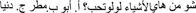 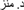  “This is Lulu. Lulu likes things that sound like her name, which of these things is the one that Lulu likes? **a.** /ʔ**ubuu**/ **‘father’** b. /mət^ʕ^ar/ ‘rain’ c. /dənya/ ‘world’ d. /mənaz/ ‘cradle’.”

### Apparatus

The experiment was designed and delivered through EyeLink 1000 Plus tracker systems (SR Research Ltd.) with a high-speed desktop-mounted 35 mm lens. Monocular eye movements were monitored and recorded with a sampling rate of 1000 Hz (1000 recordings per second). Visual stimuli were displayed on a 24″ BENQ ZOWIE XL 2430 monitor with resolution at 1920 × 1080 pixels. Participants sat at a distance of 80 cm from the screen, and their head movements were stabilized using a head-and-chin support. Audio instructions were delivered through Sennheiser HD 26 Pro noise-canceling headphones. Participants used a mouse to record their answer choices.

### Design

The behavioral tool was adapted to a computer-delivered, audio-coupled VWP. For each trial, an auditory question was introduced by a character whose name served as a prime for the target phonological condition, followed by a display showing the four possible choices arranged in a 2 × 2 grid containing the target noun and three distractors (see Figure 1). For each phonological condition, 12 test trials were presented, resulting in 36 test trials in total. In addition, at the beginning of each phonological condition block, four practice trials (not included in the analyses) were presented in order to familiarize participants with each task. Trials within each block were pseudorandomized.

### Procedure

Prior to the experiment, all participants read and signed a consent form and completed a demographic background questionnaire inquiring, amongst other things, about their linguistic history. The experiment took place in a quiet room in the Linguistics laboratory at United Arab Emirates University. Participants were asked to sit comfortably in front of the screen and adjust the height of their seat if necessary, with their chin and head on the table mounted support. A nine-point grid calibration was performed and validated for each participant before the experiment started. Participants then put on the headphones and were instructed to listen carefully to the instructions and recordings presented and make a selection, using the mouse, between the four different images appearing on the display. Each trial ended when the participant recorded their choice by left clicking on the mouse and the next trial automatically began. Participants sat through 12 trials per condition, for a total of three condition blocks. Participants were allowed breaks in between blocks, and the experiment resumed at the participant’s discretion. A central drift correction was performed before each block. Participants were debriefed by the experimenter at the end of the experiment and were allowed to ask questions. On average, each session lasted about 20 min.

### Measures

For all participants, the following measures were collected and later analyzed: Accuracy, Average Fixation Count, Average Fixation Duration, and Average Visits (Runs) to Region of Interest. A brief definition of these terms is outlined below.

#### Accuracy

The accuracy measure is the percentage of correct responses (i.e., correctly selecting the target) for each trial, automatically calculated for each participant by the Data Viewer software and then averaged across participants.

#### Average Fixation Count

The average fixation count is the average number of fixations made to the target in each trial, automatically calculated for each participant by the Data Viewer software and then averaged across participants. Individual fixations were defined as periods where the eyes were maintained stable and the saccade amplitude of eye movements did not exceed 0.0 degree per second. Fixations spanning 1 degree or less were automatically merged together by the Data Viewer software.

#### Average Fixation Duration

The average fixation duration is the average length in milliseconds (ms) of individual fixations made to the target in each trial, automatically calculated for each participant by the Data Viewer software and averaged across participants.

#### Average Visits (Runs) to Region of Interest

The average visits (runs) to region of interest is the number of individual visits to the target image. That is, the number of times participants looked at the quadrant containing the target, also called Region of Interest (ROI), left that particular ROI and then re-entered the pre-defined region, automatically calculated for each participant by the Data Viewer software and averaged across participants.

Fixations were included in the analysis only if they fell inside one of the four quadrants of the 2 × 2 grid. Any fixations falling outside of this region, or within close proximity to the grid outlines were discarded from the analyses.

## Results

A multivariate analysis of variance (MANOVA) was performed to test the effect of the three phonological conditions (OCM, *SIC*, and RM) as within-subjects variables and feedback group (Feedback vs No Feedback) as the between-subjects variable on three eye movement measures: average fixation duration, average fixation count, average ROI runs, and accuracy. We found significant main effects of Phonological condition, *F*(8,15) = 14.56, *p* < 0.001, Wilk’s Λ = 0.114, partial η^2^ = 0.886. There was no main effect of feedback group, *F*(4,19) = 0.364, *p* = 0.823, and the interaction between phonological condition and feedback failed to reach statistical significance, *F*(2,15) = 2.18, *p* = 0.092.

Univariate tests show significant differences in means due to phonological condition for average fixation duration *F*(1,22) = 14.38, *p* = 0.001, average number of fixations *F*(1,22) = 14.39, *p* = 0.001, average runs to the target *F*(1,22) = 8.07, *p* = 0.009, and response accuracy *F*(1,22) = 26.55, *p* < 0.001; the details of which were further analyzed as summarized below.

The MANOVA was followed up by *post hoc* pairwise comparisons for each measure with Bonferroni corrected alpha level of 0.0167 (0.05/3). Average Fixation Duration was significantly shorter in the OCM (*M* = 294 *ms*) and *SIC* (*M* = 284 *ms*) conditions, both *p*s = 0.003, in comparison to the RM condition (*M* = 332 *ms*).

Average Fixation Count was significantly shorter in the OCM (*M* = 1.8, *p* = 0.002) and *SIC* conditions (*M* = 1.8, *p* = 0.003) in comparison to RM condition (*M* = 2.8).

Average Visits (Runs) to Region of Interest were significantly lower in the OCM (*M* = 1.0, *p* = 0.013) and *SIC* condition (*M* = 1.1, *p* = 0.028) compared to the RM condition (*M* = 1.3).

Finally, Accuracy was significantly higher in the OCM (*M* = 98.7%) and *SIC* conditions (*M* = 96.7%, both *ps* < 0.001) in comparison to the RM condition (*M* = 75.5%).

No significant differences were found between the OCM and *SIC* conditions across all measures (*p*s > 0.05).

Table 2 below summarizes the means and standard deviations for each of the aforementioned measures across the three phonological conditions.

**TABLE 2:** Summary of means along with standard deviations of eye-tracking and behavioral data for the three conditions.

		Phonological Condition					
		OCM		*SIC*		RM	
Measures	*N*	*M*	*SD*	*M*	*SD*	*M*	*SD*
**Eye-tracking**							
Fixation duration (*ms*)	36	294	32.3	284	31.2	332	54.9
Fixation count	36	1.8	0.35	1.8	0.36	2.8	1.27
Target Runs	36	1.0	0.11	1.1	0.09	1.3	0.44
**Behavioral**							
Accuracy (*%*)	36	98.7	2.25	96.7	3.74	75.5	17.7

*M, Mean; SD, Standard Deviation; OCM, Onset Consonant Matching; SIC, Segmentation of Initial Consonant; RM, Rhyme Matching.*

## Discussion

The goal of this study was twofold. First, we wanted to test the transferability of the behavioral tool which was developed specifically to test the phonological awareness skills of EA speaking children on EA speaking adults. The assessment is performed on the speakers’ native dialect, which is the language they first acquire. Second, the tool was adapted into a computer-delivered VWP using the eye-tracking methodology where participants’ eye positions could be tracked automatically as an indicator of allocated attention. This allows us to test the validity of using eye movements as an implicit indicator of phonological awareness skills. Our final goal, not presented here, but projected for future directions, was to transfer this computer-based experiment with children.

Our hypotheses were as follows:

(1) Fixation indices of eye movements should differ according to the three phonological conditions.
(2) Explicit instructions should facilitate participants’ performance on the task in comparison to implicit instructions, where we predicted that eye movements would reflect more efficient fixation patterns in the explicit OCM condition in comparison to the implicit *SIC* condition.
(3) As Arabic words consist of consonantal roots as fundamental units, we expected to see the greatest hindrance in terms of eye movements and performance for the RM condition when compared to the consonant-based OCM and *SIC* conditions.
(4) Administering feedback during practice trials should facilitate performance overall on all measures in comparison with the no feedback group.

The results we obtained confirm two of these hypotheses. First, we did find significant differences between the phonological conditions for all fixation indices (Hypothesis #1). Second, in support to Hypothesis #3, we found that participants had significantly more problems performing in the RM condition compared to the consonant conditions, where they made longer average fixations, more fixations, more re-visits to individual targets (i.e., “runs”), and their accuracy scores were significantly lower than both consonant conditions (OCM and *SIC*). However, in terms of accuracy or eye movements, we did not find any differences between the explicit OCM and implicit *SIC* conditions (Hypothesis #2). Finally, there was no difference between the feedback and no feedback groups across all measures (Hypothesis #4).

Our results show that despite full native-language competence, EA adult speakers exhibit an asymmetry of phonological awareness between consonants (onsets) and vowels (rhymes). Participants consistently performed worse on the rhyme-based RM condition in comparison to the consonant-based conditions (OCM and *SIC*). Targets in the RM condition received more, longer average fixations, more target re-visits and were less accurately identified, indicating greater task difficulty. This is an interesting finding, since there is agreement in the literature that the development of phonological awareness skills in children mirrors the hierarchical structure of the syllable, with awareness to rhymes typically preceding sensitivity to phonemes (e.g., Ziegler and Goswami, 2005; Goswami, 2010). The consensus is that children first acquire sensitivity and awareness to larger linguistic “chunks” (i.e., words) before mastering more detailed, finer-level phonological units such as syllables, onset-rhymes, and phonemes, respectively (Goswami, 2010). Our findings suggest a trend in the opposite direction, as adults performed better in tasks concerning consonants than rhymes.

However, orthographic transparency also affects the way readers acquire the phonological skills necessary when learning to read. In languages where grapheme-to-phoneme mappings are consistent in alphabetic orthographies (such as German and vowelized Arabic), sensitivity to phonemes develops quicker than in languages with inconsistent orthographies, since they are the most salient phonological units in these languages (Goswami, 2010). In the case of Arabic, consonants make up the majority of the phonemes in the language. Short vowels (shown as optional diacritics) are often left out of writing and do not typically appear in printed text. This is reflected in the current methods of literacy instruction, where children are first taught to read and write using full vowelization. However, later in primary education years, children are discouraged from using diacritics in writing except in cases where those are necessary to disambiguate homographic words (Abu-Rabia, 2002). Therefore, it is almost as though from a very young age native Arabic speakers are taught to disregard vowels at least in writing, perhaps for their redundancy in context. Instead, readers often rely on contextual clues to disambiguate words during reading (Abu-Rabia, 1997, 2012, 2019). Both vowels and context play a role in disambiguating homographs and, therefore, facilitate text comprehension (Abu-Rabia, 1997). Therefore, it was predicted and subsequently shown that Arabic speakers would show the highest efficiency in performance, as reflected by fewer, shorter average fixations and re-visits to target ROIs, for consonant-based conditions as opposed to rhyme-based (vowels) conditions.

We found no evidence to support the claim that performance will differ between the two consonant conditions. However, this lack of results may possibly be attributed to the low number of trials (12 total) in each condition group yielding low statistical power. Moreover, we could not find differences across feedback groups. Perhaps this is due to the characteristics of the sample population recruited for this study, since all participants were adults recruited from a university pool; it is highly likely that their phonological skills are well developed, such that they are able to successfully complete the given tasks without needing further instructions and without benefiting further from practice feedback. This would not be expected with a much younger sample population who will likely be at different mastery levels of phonological awareness skills.

We showed that it is possible to use eye-tracking to assess phonological awareness in EA speaking adults and demonstrated that eye movements differed depending on the phonological conditions. Our study could be used to examine EA speaking children’s phonological awareness. It is important to note that this particular study is not exhaustive of all the possible phonemes in EA, and further research is needed to test the feasibility of using this experiment design to capture the same differences between and within conditions of the full list of phoneme in EA. In addition, the current experiment could be adapted to evaluate processing of Arabic script, in order to determine the link between phonological awareness and literacy. While the current aim is to focus on the phonological knowledge of EA words which may not correspond to MSA words, further research can be conducted so that the phonological awareness of EA and MSA can be compared using the eye movement study (cf. Saiegh-Haddad, 2003).

## Data Availability Statement

The datasets generated for this study are available on request to the corresponding author.

## Ethics Statement

The studies involving human participants were reviewed and approved by Social Sciences Research Ethics Committee Human Medical Research Ethics Committee United Arab Emirates University. The patients/participants provided their written informed consent to participate in this study.

## Author Contributions

AM, MA, and TL conceived the project and planned the experiment design. The task, conditions and stimuli were selected and developed by AM and her research assistants. FB programed the study, collected data and performed statistical analyses. All authors discussed the results and contributed to the final manuscript.

## Conflict of Interest

The authors declare that the research was conducted in the absence of any commercial or financial relationships that could be construed as a potential conflict of interest.
